# An Interpretable Machine Learning Method for the Detection of Schizophrenia Using EEG Signals

**DOI:** 10.3389/fnsys.2021.652662

**Published:** 2021-05-28

**Authors:** Manuel A. Vázquez, Arash Maghsoudi, Inés P. Mariño

**Affiliations:** ^1^Department of Signal Theory and Communications, Universidad Carlos III de Madrid, Leganés, Spain; ^2^Department of Biomedical Engineering, Science and Research Branch, Islamic Azad University, Tehran, Iran; ^3^Department of Biology and Geology, Physics and Inorganic Chemistry, Universidad Rey Juan Carlos, Móstoles, Spain; ^4^Research Laboratory Systemic Medicine of Healthy Ageing, Institute of Biology and Medicine, National Research Lobachevsky State University of Nizhny Novgorod, Nizhny Novgorod, Russia; ^5^Institute for Women's Health, University College London, London, United Kingdom

**Keywords:** electroencephalography, machine learning, random forest, schizophrenia, connectivity, direct directed transfer function, generalized partial directed coherence

## Abstract

In this work we propose a machine learning (ML) method to aid in the diagnosis of schizophrenia using electroencephalograms (EEGs) as input data. The computational algorithm not only yields a proposal of diagnostic but, even more importantly, it provides additional information that admits clinical interpretation. It is based on an ML model called random forest that operates on connectivity metrics extracted from the EEG signals. Specifically, we use measures of generalized partial directed coherence (GPDC) and direct directed transfer function (dDTF) to construct the input features to the ML model. The latter allows the identification of the most performance-wise relevant features which, in turn, provide some insights about EEG signals and frequency bands that are associated with schizophrenia. Our preliminary results on real data show that signals associated with the occipital region seem to play a significant role in the diagnosis of the disease. Moreover, although every frequency band might yield useful information for the diagnosis, the beta and theta (frequency) bands provide features that are ultimately more relevant for the ML classifier that we have implemented.

## 1. Introduction

Schizophrenia is a severe mental disorder that compromises significantly many aspects of the quality of life and affects more than 20 million people worldwide (Insel, 2010). Due to the absence of validated and reliable biological markers, diagnosis of schizophrenia is mostly subjective and mainly based on documented symptoms (such as hallucinations, disorganized speech, etc.), their duration, or apathy at work and/or social activities (see Segal, 2010 for a thorough review). While schizophrenia is known to have an effect on the activity of the brain (Rubinov et al., 2009), other mental disorders such as, e.g., obsessive compulsive disorder, attention deficit hyperactivity disorder, or bipolar disorder produce similar variations in the baseline brain activity (Anier et al., 2012). Moreover, mental diseases such as bipolar disorder or major depressive disorder are often confused with schizophrenia. Thus, finding automatic tools to help clinicians in the diagnosis of the disease is a challenging problem.

In recent years machine learning (ML) techniques have become important tools in addressing classification tasks that involve medical problems. As examples, we can mention the use of long short-term memory recurrent neural networks (RNNs) to classify diagnoses from pediatric intensive care unit data (Lipton et al., 2015), the use of RNNs and Bayesian models to discriminate patients with ovarian cancer (Mariño et al., 2017; Vázquez et al., 2018), the use of support vector machines (SVMs) for attention deficit hyperactivity disorder prediction (Dai et al., 2012), the application of convolutional neural networks (CNNs) to classifying electroencephalogram (EEG) signals for emotion recognition (Luo et al., 2020), or the combination of multilayer perceptrons and SVMs to diagnose major depressive disorders (Saeedi et al., 2020b).

In this work we contribute to this mainstream of research by proposing an ML method to aid in the diagnosis of schizophrenia using EEGs as input data. This type of signals has been extensively used in the literature for non-invasive studies of the brain electrical activity (Asadzadeh et al., 2020), including classification of several mental disorders as dementia (Durongbhan et al., 2019), depression (Acharya et al., 2018; Saeedi et al., 2020a) and schizophrenia (Sabeti et al., 2011; Thilakvathi et al., 2017; Shalbaf et al., 2020; Chandran et al., 2021).

Current research in the literature for schizophrenia classification is rooted in black-box models that fail to provide transparency for clinicians (Sabeti et al., 2011; Thilakvathi et al., 2017; Shalbaf et al., 2020; Chandran et al., 2021). While such methods may attain good classification performance on validation data sets, they yield no “justification" of their outputs (i.e., these outputs bear no interpretable features). Clinicians should be able to judge how automatic classifications are made, and choose how to use that information in combination with their own examination and training. Some attempts at interpretability in the field of discrimination of patients with schizophrenia have been carried out very recently by using magnetic and structural magnetic resonance imaging (MRI) (de Pierrefeu et al., 2018; Reiter, 2020). Similarly, Acar et al. (2017) have proposed a technique based on tensor decompositions for the identification of event related potentials (ERPs) in functional MRI data and EEG recordings that may be indicative of schizophrenia.

The computational technique proposed in this paper not only yields a proposal of diagnosis but, even more importantly, it provides additional information that admits clinical interpretation. Specifically, the proposed approach is based on an ML model called random forest (Breiman, 2001) that operates on connectivity metrics extracted from EEG signals. Random forests allow assessing which input features are most relevant to the classification task, which is the reason why they are specially appealing for the problem at hand. Input features to this ML model are given by measures of generalized partial directed coherence (GPDC) (Baccalá and Sameshima, 2001; Baccala et al., 2007) and direct directed transfer function (dDTF) (Kamiński et al., 2001) computed from the EEG signals. The studies we have conducted using the outlined methodology allow to identify EEG signals (and frequency bands) that might play a key role in revealing important information about the physiology of the brain in schizophrenia patients.

The paper is organized as follows. In Section 2 we describe the dataset used in this manuscript as well as the necessary pre-processing. The random forest algorithm is presented in Section 3. In Section 4 we describe the two data analysis carried out in this work, and Section 5 is devoted to a discussion of the results.

## 2. Data pre-processing

### 2.1. Raw Data

**Figure 1:**
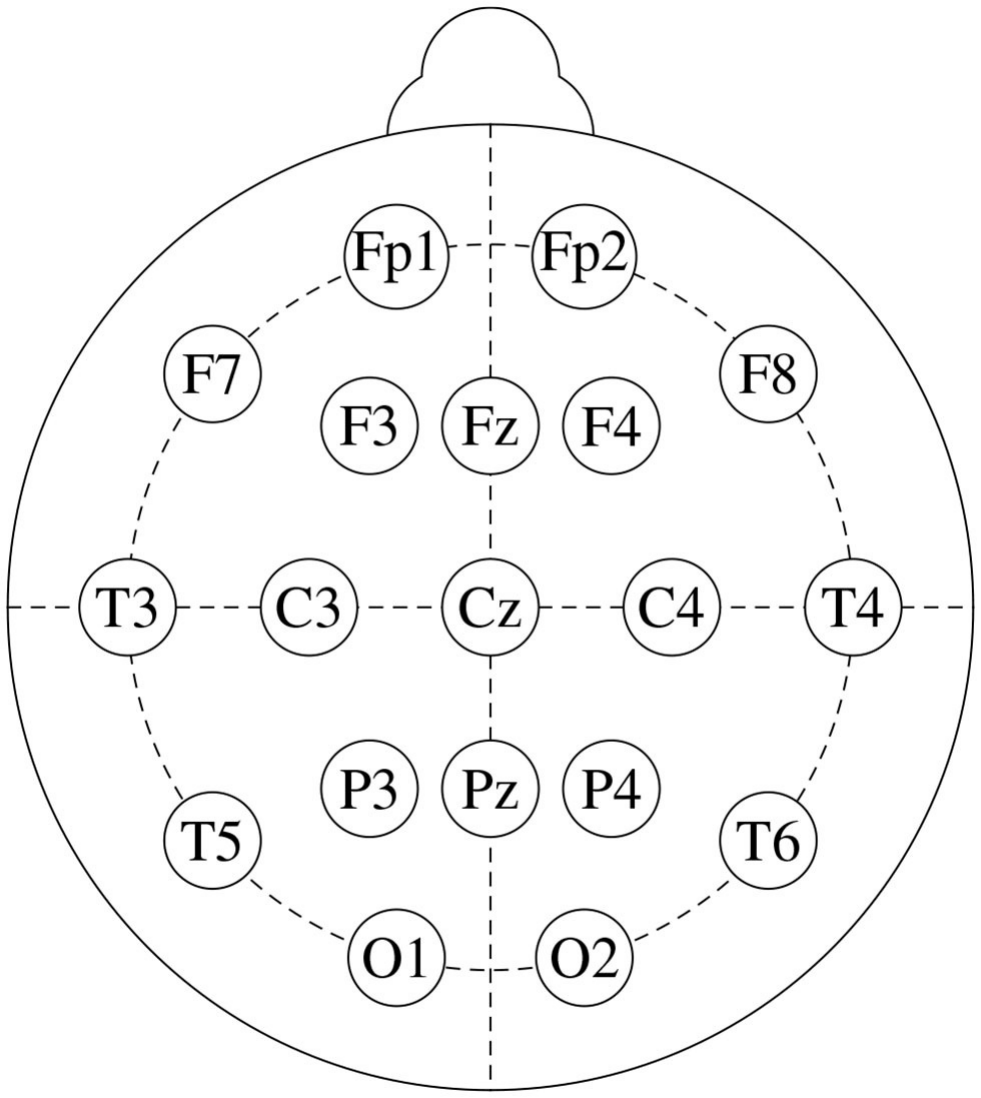
Standard 10-20 EEG setup, that consists of 19 channels: Fp1, Fp2, F7, F3, Fz, F4, F8, C3, Cz, C4, P3, Pz, P4, T3, T4, T5, T6, O1, and O2.

We base this study on freely available data from a public repository^1^. It consists of EEG recordings from 14 patients suffering from paranoid schizophrenia (7 males: 27.9±3.3 years old, and 7 females: 28.3±4.1 years old) and 14 age-matched healthy controls (7 males: 26.8±2.9 years old, and 7 females, 28.7±3.4 years old). Signals were recorded for 12 minutes, with subjects in closed-eyes state, with a sampling frequency of 250 Hz. Figure 1 shows the standard 10-20 EEG setup that was used to record the data. It yields 19 channels per subject: Fp1, Fp2, F7, F3, Fz, F4, F8, C3, Cz, C4, P3, Pz, P4, T3, T4, T5, T6, O1, and O2. For further details on the dataset, see Olejarczyk and Jernajczyk (2017).

From the latter reference, and regarding the use of data coming from human subjects, it is important to emphasize that the “study protocol was approved by the Ethics Committee of the Institute of Psychiatry and Neurology in Warsaw,” and “all participants received a written description of the protocol and provided written consent to take part” in the corresponding study.

### 2.2. Effective Connectivity Estimation

Effective connectivity has become a prevalent analysis tool in current neuroimaging since it is able to explore causal effects between different brain areas and determine directionality of neural interactions (Astolfi et al., 2007). The most popular strategy for connectivity estimation is Granger causality (GC) which is a model-based methodology (Granger, 1969). In the event that a signal *x* can be estimated by previous data from another signal *y* in a way that is better than from its own data, then the signal *y* is viewed as the cause for the primary signal *x*. GC measures can be obtained in the frequency domain, which permits the investigation of EEG recurrence in different bands (Geweke, 1984). In order to accomplish this, a multivariate autoregressive (MVAR) model of each individual signal is required.

Let *x*_*t*_ be a vector representing an *m* channels signal at time *t*. The MVAR model is written as

(1) $$\begin{array}{r} x_{t}=\overset{p}{\underset{k=1}{\sum }}A_{k}x_{t-k}+u_{t}, \end{array}$$

where *p* denotes the model order, *A*_*k*_ is an *m* × *m* matrix, and *u*_*t*_ is an *m*×1 (column) vector of white noise with covariance matrix C. By rearranging terms, Equation (1) can be written as

(2) $$u_{t}=\sum_{k=0}^{p}{\hat{A}}_{k}x_{t-k,}\text{with }{\hat{A}}_{k}=\left\{ \begin{array}{l} -A_{k},\;k=1,...,p\\ I_{p},\;k=0\\ 0,\text{ otherwise } \end{array}\right.$$

where *I*_*p*_ is the identity matrix of order *p*. The summation on the right-hand-side of Equation (2) is a convolution sum, and hence taking the Fourier transform on both sides of the equation, we have

(3) $$\begin{array}{r} U\left(f\right)=A\left(f\right)X\left(f\right), \end{array}$$

where *U*(*f*) and *X*(*f*) are the spectral representations of vectors *u*_*t*_ and *x*_*t*_, respectively, and

(4) $$\begin{array}{r} A\left(f\right)=\overset{p}{\underset{k=0}{\sum }}{\hat{A}}_{k}e^{-2\pi fk\sqrt{-1}}. \end{array}$$

Solving for *X*(*f*) in Equation (3) results in

(5) $$\begin{array}{r} X\left(f\right)=A{\left(f\right)}^{-1}U\left(f\right). \end{array}$$

For the sake of conciseness we define the *m* × *m* matrices

(6) $$\begin{array}{r} H\left(f\right)=A{\left(f\right)}^{-1}\\ S\left(f\right)=X\left(f\right)X^{*}\left(f\right), \end{array}$$

which along with *A*(*f*) can be exploited to compute various effective connectivity measures. Two common quantitative spectral measures are GPDC (Baccalá and Sameshima, 2001; Baccala et al., 2007) and dDTF (Kamiński et al., 2001), which are defined between channels *i* and *j* as, respectively,

(7) $$\begin{array}{r} {\text{GPDC}}_{ij}\left(f\right)=\frac{\frac{1}{{\text{C}}_{ii}}A_{ij}\left(f\right)}{\sqrt{\sum_{k=1}^{m}\frac{1}{{\text{C}}_{kk}}|A_{kj}\left(f\right){|}^{2}}} \end{array}$$

and

(8) $$\begin{array}{r} {\text{dDTF}}_{ij}\left(f\right)=\frac{|H_{ij}\left(f\right){|}^{2}}{\sum_{f}\sum_{k=1}^{m}|H_{ik}\left(f\right){|}^{2}}\frac{S_{ij}^{-1}\left(f\right)}{\sqrt{S_{ii}^{-1}\left(f\right)S_{jj}^{-1}\left(f\right)}}, \end{array}$$

where, for a matrix *A*, we denote as *A*_*ij*_ the element in the *i*-th row and the *j*-th column. Both GPDC and dDTF try to describe the causal relationship between a pair of signals (coming from the EEG, in our particular case), say *i* and *j*. GPDC puts the focus on signal *i* as a source (producing a flow of information), whereas dDTF is concerned with signal *j* considered as a sink (receiving the flow).

We have computed both connectivity measures, GPDC_*ij*_(*f*) and dDTF_*ij*_(*f*), for every possible combination of EEG channels (*i, j* = 1, ⋯ , *m, i* ≠ *j*), and frequency bands:

- delta (1-4 Hz),
- theta (4-8 Hz),
- alpha (8-12 Hz),
- beta (12-30 Hz), and
- gamma (30–50 Hz).

**Figure 2:**
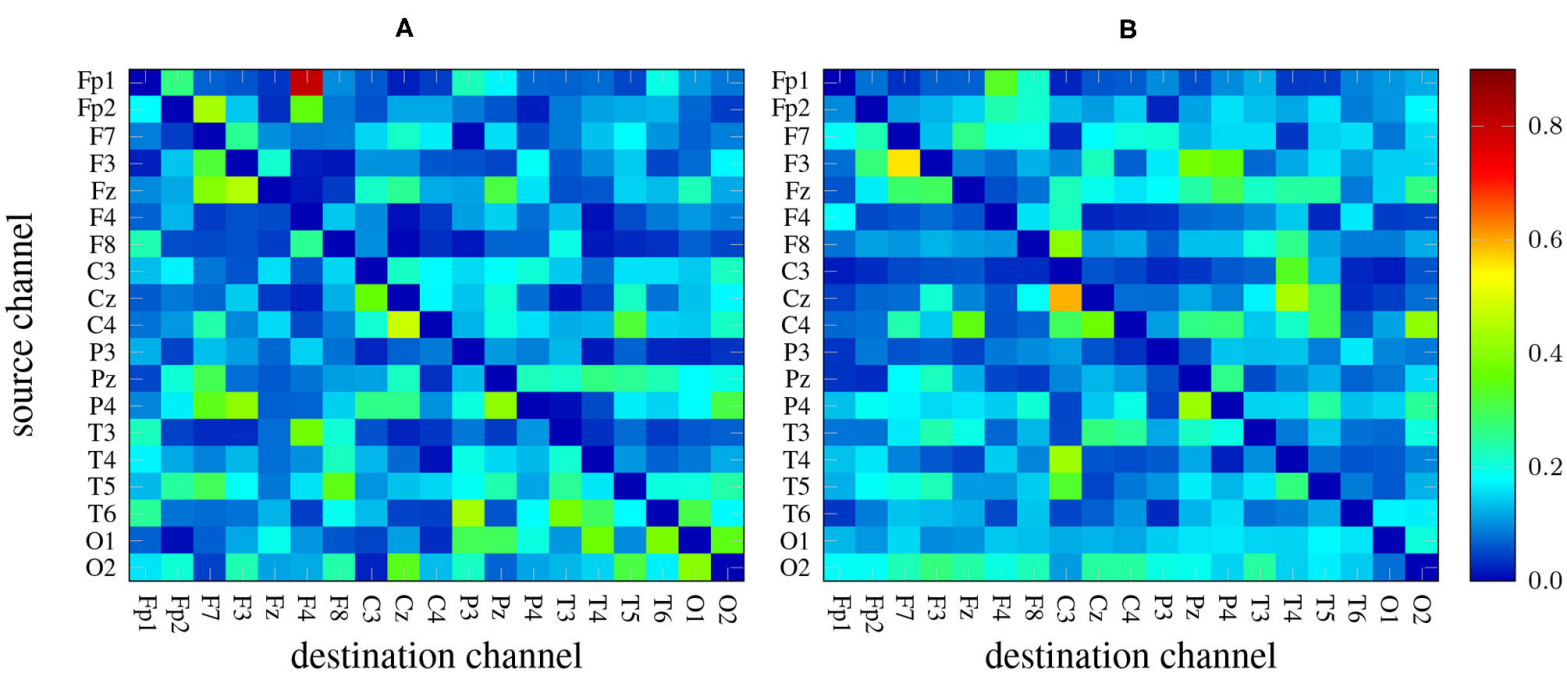
GPDC samples computed in the alpha band of a 1-minute EEG recording coming from a patient **(A)** and from a healthy subject **(B)**. Red color indicates high values whereas dark blue color represents lower values.

There are 19 × 19−19 = 342 channel combinations^2^ that, along with the 5 frequency bands yield an overall number of 1710 *features* per measure. As an example, Figure 2 depicts two heat maps with the values of GPDC (for every pair of channels) computed in the alpha band of a 1-minute EEG segment coming from a patient (left) and a healthy subject (right). Notice the values in the diagonal are, in any case, all zero.

### 2.3. From Raw EEG to Samples

Raw signals were processed with low- and high-pass Butterworth filters with cut-off frequencies of 0.5 and 50 Hz, respectively. Afterwards, the EEG recording of every subject (encompassing the 19 channels) was split into 1-minute windows (signal segments). An MVAR model with *p* = 5 was then fitted (see Equation (1)) to every individual window. From the latter, Equations (7), (8) allow computing the GPDC and dDTF metrics, respectively, between any given signals, *i* and *j*, and for any frequency of interest, *f*. Getting the value of a metric for a certain frequency band (as opposed to a particular frequency, *f*) involves evaluating the metric at a sequence of frequencies covering the corresponding interval and computing the average. In our experiments, this sequence is spanned from the lower to the upper bounds of the interval by increments of 1 hertz. The length of the window used to split the signals into samples (1 minute) and the order of MVAR model fitted to the EEG signals (*p* = 5) were selected using the autocorrelation function and portmanteau tests.

Ultimately, all the EEG recordings are split into 644 segments, and this is the overall number of samples. Each one of them encompasses features from both GPDC and dDTF connectivity measures, and hence has size 3420.

## 3. Materials and Methods

**Figure 3:**
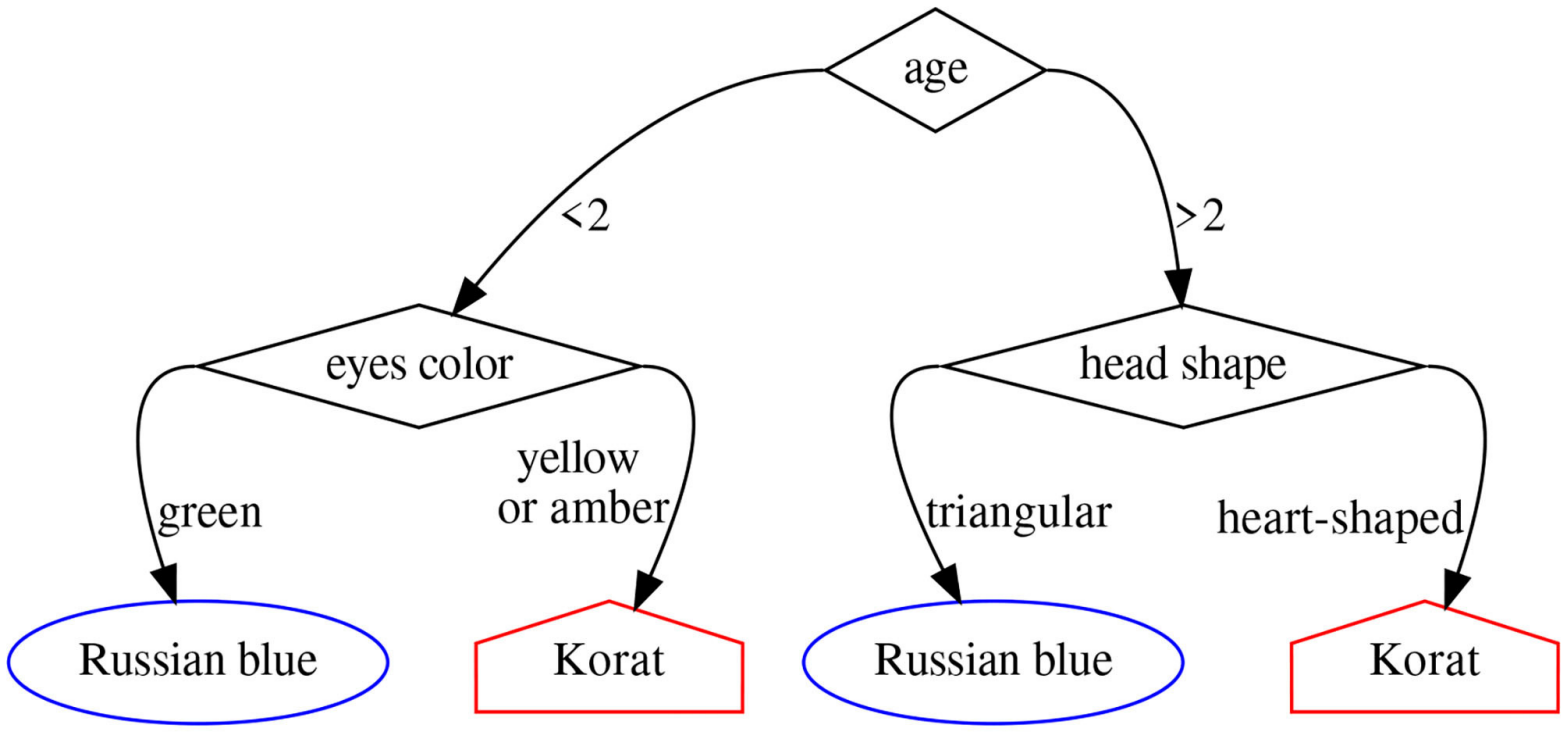
Decision tree to determine whether a cat belongs to the *Russian blue* or the *Korat* breed.

A random forest (Breiman, 2001) is simply an *ensemble* of decision trees, each one trained on a different subset of the data and the available features. A decision tree (Magee, 1964), in turn, is a modeling approach based on splitting a collection of data points into mutually exclusive groups by asking a series of binary *yes-or-no* questions. Each group or *leaf* in the resulting tree is then assigned an outcome (a number) in a regression problem, or a label in a classification one. Figure 3 shows an example of decision tree to tell apart the *Russian blue* and *Korat* cat breeds.

Training a decision tree involves finding, at every step of the algorithm, the best split of the data according to some prescribed metric. The process starts with all the samples at the *root* of the tree, and proceeds recursively splitting the samples at every leaf node (in the beginning just the root) into two children nodes until a certain stopping criterion is met. At every step of the algorithm, in order to find the best split for a node, we need to compare the results that would be obtained using every possible combination of feature and value thereof. This comparison is carried out using the metric of choice, which in our case is the Gini index (Breiman et al., 1984). The latter yields a measure of the *impurity* of a group of samples according to the classes they belong to. Specifically, if in a binary classification problem a certain node has *p*_*A*_ percentage of samples from class *A* and *p*_*B*_ = 100−*p*_*A*_ percentage from class *B*, then the Gini impurity of that node is given by

(9) $$\begin{array}{r} G=p_{A}\left(1-p_{A}\right)+p_{B}\left(1-p_{B}\right). \end{array}$$

When evaluating a split for a node, each child will have its own Gini index, and a measure of the latter for the parent node can be computed as a weighted average of those from the children, each one multiplied by the percentage of samples from the parent node that it contains after the split.

The training algorithm is greedy in the sense that, at every step, the best split is selected for each node (that must be further split according to the stopping rule), and no *backtracking* is later performed. The stopping rule is a hyperparameter, and a common choice is to not further split leaf nodes whose number of samples is below a certain threshold (Ranganathan et al., 2018). We abide by this criterion here.

As mentioned above, a random forest is simply a collection of decision trees that are trained on different subsets of the same dataset. They are based on the idea of *bagging predictors* (Breiman, 1996), which consists in constructing different versions of a classifier (a predictor) each one trained on a different *bootstrap replicate*^3^ of the training set. Since the classifiers are trained on different datasets, the errors they make are (approximately) uncorrelated, and hence their average is 0 (assuming every individual classifier is working properly and its expected error is also 0).

Bagging is a very general procedure in machine learning that can be, in principle, applied to many different kinds of predictors. In random forests, *bootstrap replicates* are obtained from the training set by subsetting both dimensions in the data: the *sample dimension* and the *feature dimension*. In other words, each version of the classifier is trained on a subset of randomly selected samples, using only a subset of randomly selected features.

One appealing feature of random forests is that, after training, they allow computing a measure of *importance* for every feature that indicates how much it contributed to the classification process. This is achieved by exploiting the Gini index in a slightly different way. For a certain feature in a given decision tree, Gini *importance* is computed by adding up the *decrease* in the Gini index that is attained every time a split on that feature takes place. In a random forest, we must account for this metric in all the trees in the ensemble (see Breiman et al. (1984) for details). Although other (equally performing) measures of importance are possible, we rely on the Gini index because it is readily available in most ML software libraries.

## 4. Results

Random forests often require very little tuning (see e.g., Hastie et al., 2009). In this particular case, each random forest encompasses *T* = 200 individual decision trees in which the minimum number of samples per leaf is *M* = 10. Every decision tree is built on only 85 randomly selected features, which is around *P* = 2.5% of the *N* = 3420 overall number. The hyperparameters *T*, *M*, and *P* have been selected empirically after a few preliminary experiments.

Since (after pre-processing) the number of samples is relatively small (only 644), we use *K*-fold cross-validation to get a more accurate assessment of the classifier's performance. Hence, we split the data into *K* equal-sized disjoint sets (also known as folds), and separately (in turns) evaluate the performance on each one while training on the rest. We apply this strategy with *K* = 7 in two different ways.

### 4.1. Subjet-Unaware Partitioning

We first split the samples into training and test sets ignoring the subject from which each sample originates. Therefore, this becomes a regular binary classification problem in which each sample is labeled as “coming from a healthy subject” or “coming from a patient”. In order to implement this strategy, we carry out the training-test split within each subject (according to the ratio determined by the number of folds) and then the training sets from all the individual subjects are stacked together to yield the (overall) training set, and an analogous procedure is used to construct the (overall) test set^4^.

**Figure 4:**
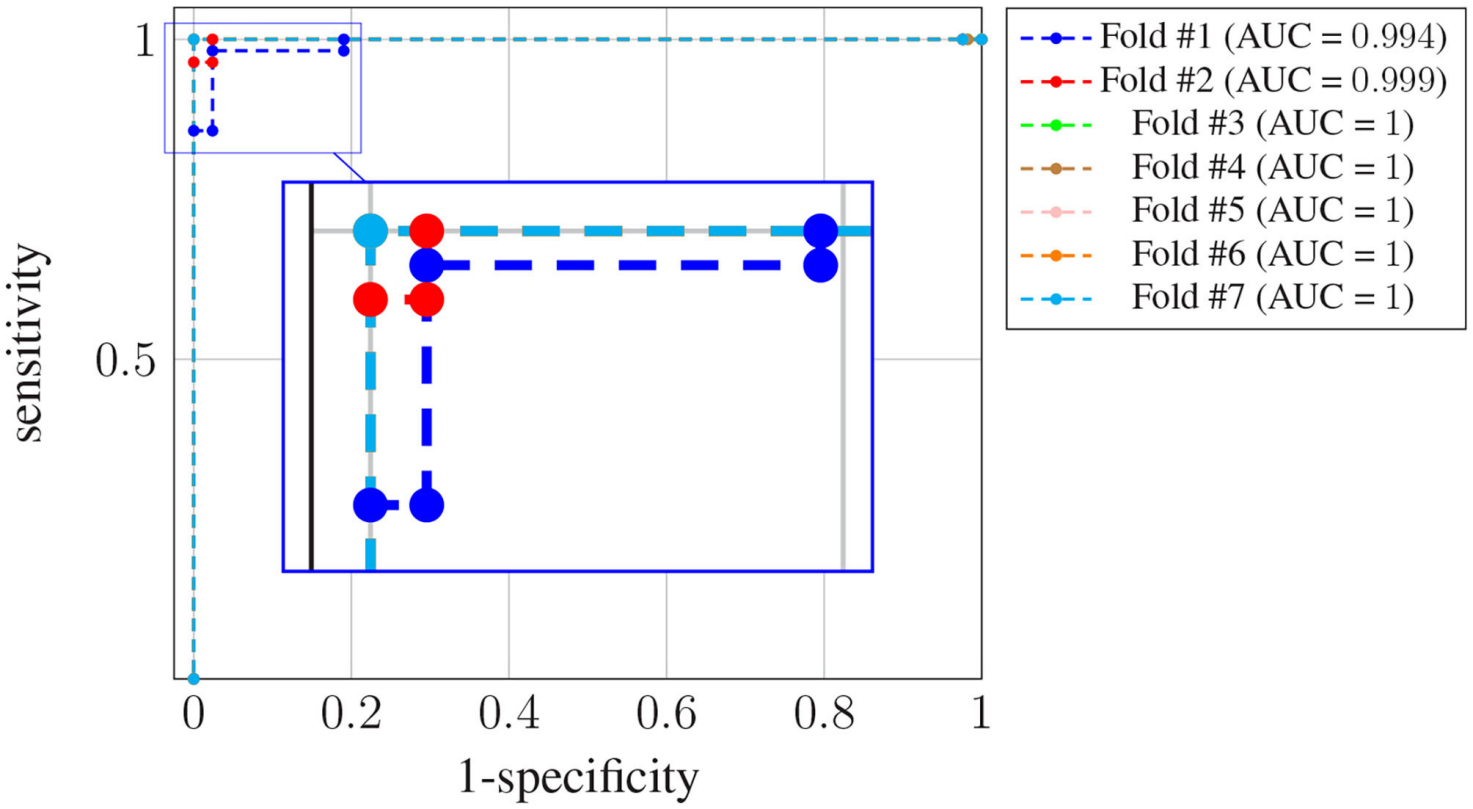
ROC curves for subject-unaware data partitioning strategy. Each curve corresponds to a different fold, and the attained AUC is, in every case, shown in the legend.

Figure 4 shows the receiver operating characteristic (ROC) curve for each one of the *K* = 7 folds.

It can be seen that, in every case, a sensitivity very close to 1 can be attained even for large values (also close to 1) of the specificity (notice the horizontal axis is not specificity but its complementary). Also, the difference in the ROC curves of the different folds is negligible, and confined to very large values of the specificity. The zoom box in the figure attempts to make this difference noticeable. The legend next to the plot also shows the area under the curve (AUC) attained for every fold. On average it is above 0.99.

Since we have different results of *feature importance* for different folds, we are going to use the minimum and the average as summary statistics. Specifically, the minimum importance is used to assess whether a certain feature was consistently important across all the folds, whereas the average importance is used to aggregate the results from all the folds. If we let *Iji* denote the importance of the *i*-th feature in the *j*-th fold, then the minimum importance of the *i*-th feature is

(10) $$I_{i}^{\min}=\min{\left\{I_{i}^{j}\right\}}_{j=1}^{K}$$

while the average importance is

(11) $$I_{i}^{\text{avg}}=\frac{1}{K}\sum_{j=1}^{K}I_{i}^{j}.$$

Notice that the importance of every feature is normalized so that they all add up to 1, and hence the importance of a given feature provides information about its *relative* importance as compared to others.

**Figure 5:**
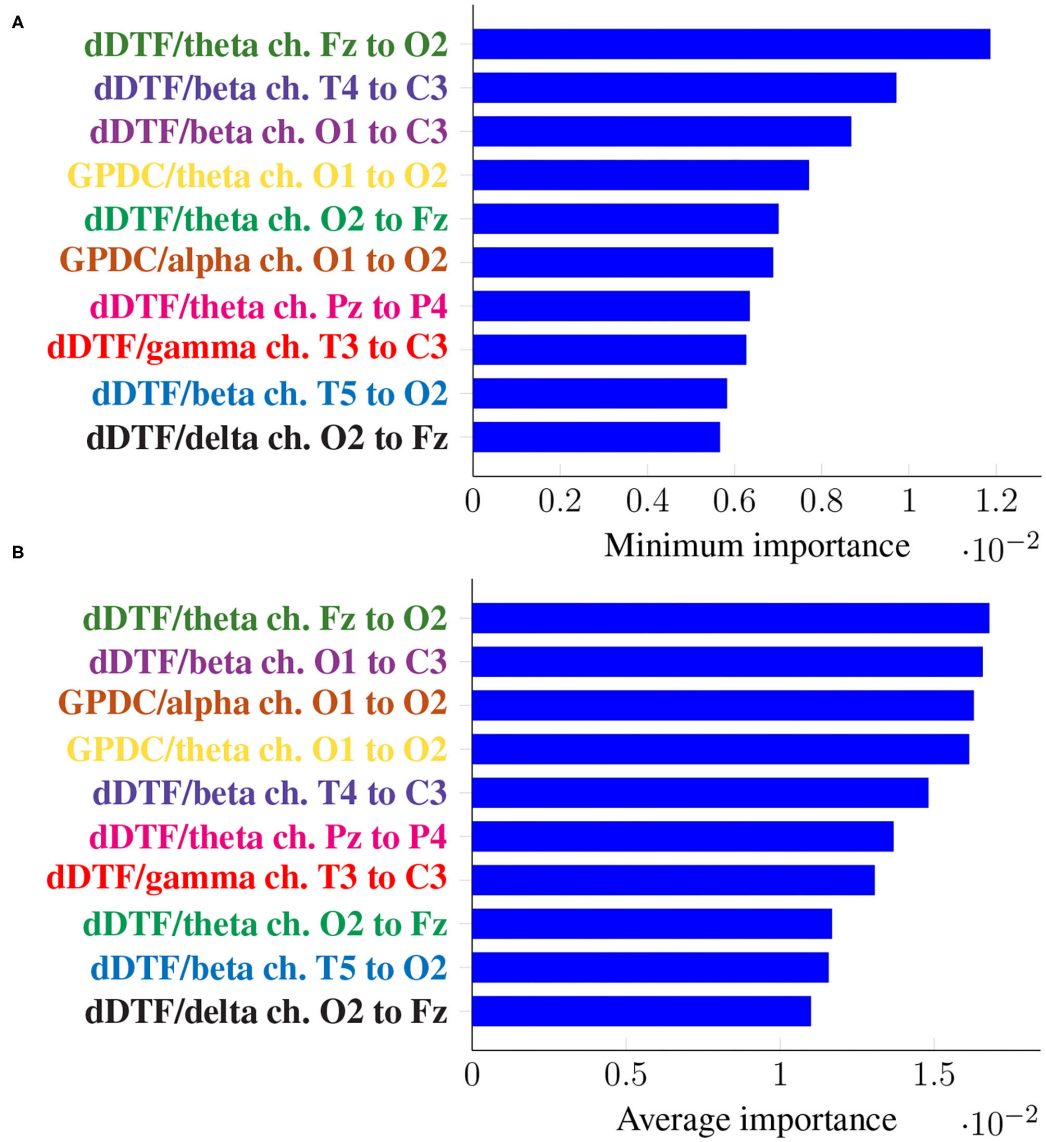
Feature importance summarized across folds using the minimum **(A)** and the mean **(B)** for subject-unaware data partitioning strategy. Every feature is determined by the metric (left of “/”), the frequency band (right of “/”), and the EEG channels involved (notice the order is important).

Figure 5 shows the top 10 features when these are ranked (in descending order) according to their corresponding *I*min, in the top panel, and *I*avg in the bottom one. Every feature name is colored differently but consistently across panels for easier comparison.

It can be seen that the exact same 10 features are present in both panels, meaning not only they have a large importance on average, but they are consistently important across folds (i.e., it is not the case that they are very important in a certain fold and not at all in the rest). Moreover, some features are ranked at exactly the same place in both panels of Figure 5.

### 4.2. Leave-p-Subjects-Out

A more realistic assessment of the model performance entails building the training and test sets while accounting for the subject originating every sample. The motivation behind this is that, in a real-world scenario, we usually want to exploit the classifier in deciding whether or not a *new* subject (never seen before) is or not affected by the disease. In order to emulate this scenario in our evaluation of the model we can use a leave-p-subjects-out strategy, which dictates that the classifier must be validated in subjects that are not part of the training set. This is again implemented with a 7-fold cross-validation, but in this case at the subject level: every fold comprises the samples of 2 healthy subjects and 2 patients. Thus, the model is trained each time on 24 subjects (12 healthy ones and 12 patients), and evaluated on 4 *different* ones.

**Figure 6:**
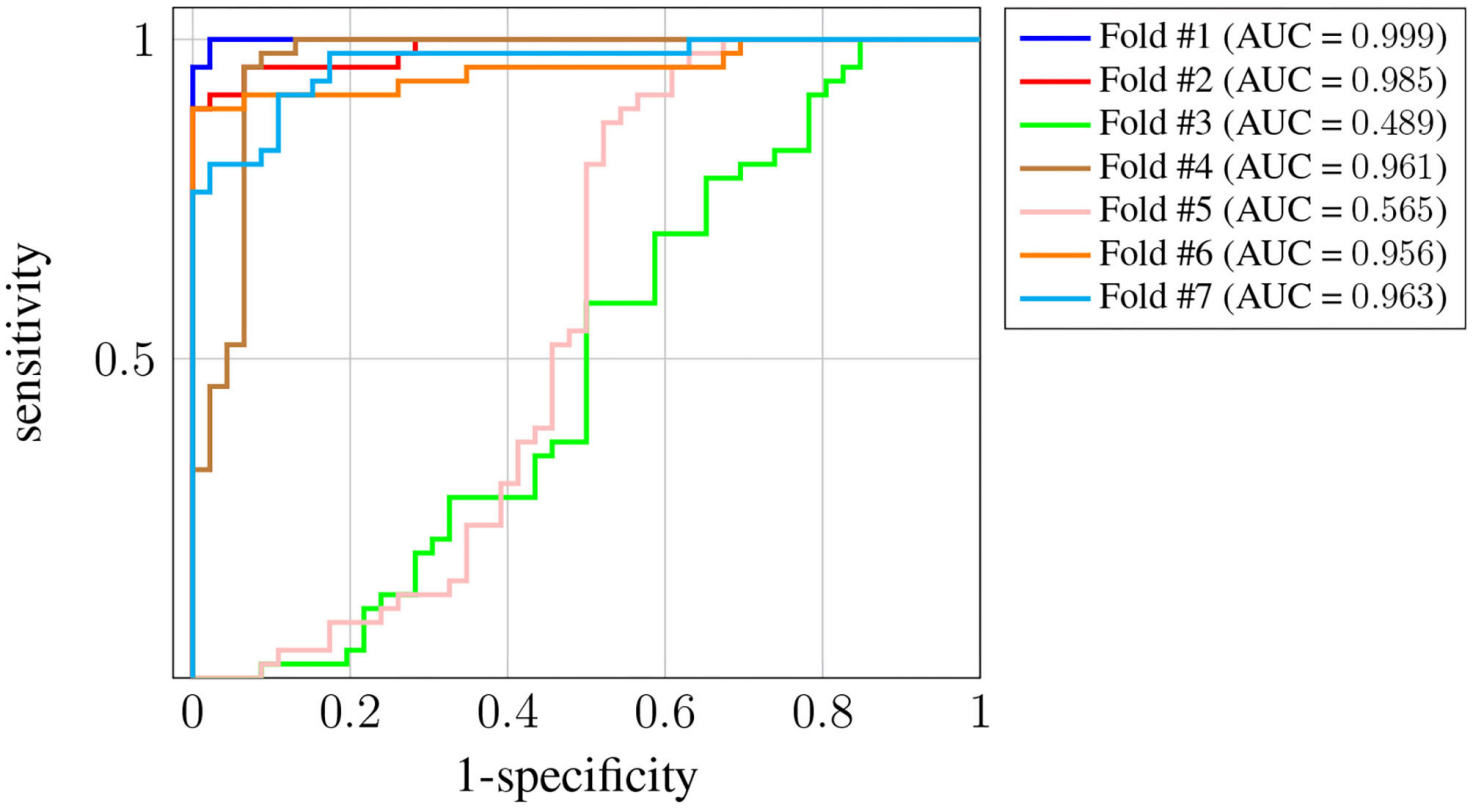
ROC curves for Leave-p-Subjects-Out cross-validation. Each curve corresponds to a different fold, and the attained AUC is, in every case, shown in the legend.

Figure 6 shows the ROC curve for each one of the *K* = 7 folds. In this case we have more variance across the different folds, and the AUC for two of them (#3 and #5) is significantly worse^5^. Nevertheless, the AUC is still above 0.95 for the rest of the folds, and the average is around 0.87. The decrease in the performance obtained when using this last strategy suggests there is significant subject-to-subject variation. This issue might be addressed by way of a user-specific calibration of the classifier, as it is sometimes done in brain-computer interface systems [see, for instance, Wilson et al. (2009)]. However, such approach falls out of the scope of this work.

**Figure 7:**
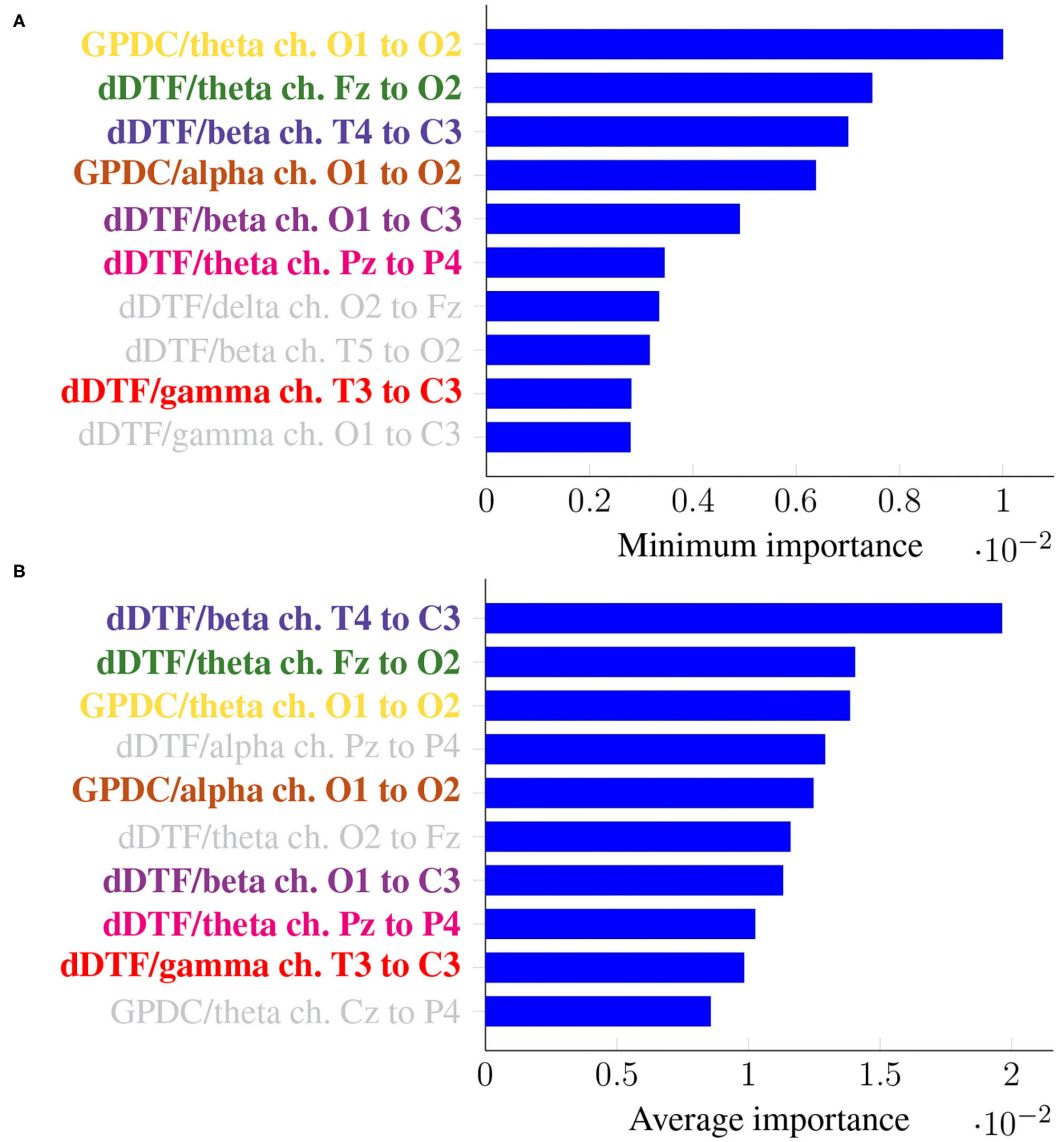
Feature importance summarized across folds using the minimum **(A)** and the mean **(B)** for Leave-p-Subjects-Out cross-validation. Every feature is determined by the metric (left of “/”), the frequency band (right of “/”), and the EEG channels involved (notice the order is important).

As before, it is of interest to identify features that are consistently relevant for classification. Figure 7 summarizes feature importance in the same way we did in the previous section.

In this case, the two rankings fail to exhibit the exact same features, but many features with large minimum importance across folds also attain a large average importance. Furthermore, many of the features that were relevant in the previous section are still relevant here (for easy matching, colors are consistent in both Figures 5, 7). Features that are unique to a single panel are grayed out.

Notice that in both this section and Section 4.1, evaluation is carried out using a 7-fold cross-validation strategy, entailing a *test* set that encompasses 100/7 ≈ 14% of the samples, which seems a sensible choice given the relatively small size of the dataset.

## 5. Discussion

In this work we have tackled the problem of assessing whether a subject suffers from schizophrenia or not by analyzing their recorded EEG signals. We compute effective connectivity measures on the latter that become the input features of a random forest. This ML technique allows to interpret the results of the classifier by identifying those features that are most relevant for performance. Thus, we have selected seven features (highlighted in color in Figure 7) that in our analysis seem to play a role in telling apart patients from healthy subjects.

Attached to every selected feature is a connectivity measure (either GPDC or dDTF), a frequency band, and a pair of channels that are causally related. Hence, a method like the one proposed in this work can help clinicians locate key areas and/or connections in the brain that are related to schizophrenia. For instance, our results suggest that signals O1 and O2 are important, specially in the theta and alpha bands. These two signals are associated with the occipital lobe region, and a link between the latter and schizophrenia has already been established before in the literature (see e.g., Onitsuka et al., 2007; Tohid et al., 2015). At the sight of Figure 7, and given that signal C3 shows as the *sink* in three different features, the central lobe of the brain also seems to play a prominent role in the disease. Some other conclusions can be drawn from the same figure. However, and due to the relatively limited sample size, we reckon this is a pilot study and further research, with a larger sample size, would be needed to validate and, afterwards, properly interpret the preliminary results reported here. When comparing our results with those in Olejarczyk and Jernajczyk (2017) (where the dataset was originally studied) there are some similarities. Although the work in the aforementioned paper is based on an entirely different method (relying on graph analysis), it also evinces, for instance, the importance of the occipital area in the alpha band.

Regarding the frequency bands, there are many studies supporting the influence of the alpha band, as well as delta and theta. A thorough review of many of them is carried out in Newson and Thiagarajan (2019). The authors of the latter claim that findings regarding schizophrenia are mostly coherent, though there are some inconsistencies. Therefore, this is still an open problem that should be further pursued.

With respect to the evaluation of the method, we remark that performance can be very different depending on whether or not the classifier is evaluated on samples originating from subjects that have been seen during training. If we guarantee that a few samples from each subject are included in the training set, then the average AUC is 0.99, whereas if subjects in the training and test sets are different, that number decreases down to 0.87. Nevertheless, in this work the focus is on the interpretability of the decisions, and we have found that, in any case, some common conclusions can be drawn.

As noted in Section 1, there are other mental disorders that affect EEG in a similar way as schizophrenia does, and hence can be easily confused with it. A relevant line of future research is to address the question of whether the proposed ML approach is useful in separating schizophrenia from other mental diseases producing similar variations in the EEG.

## Data Availability Statement

Publicly available datasets were analyzed in this study. This data can be found here: https://doi.org/10.18150/repod.0107441.

## Author Contributions

MV and IM conceived the idea. AM performed data pre-processing. MV designed and performed the data analysis. All authors contributed to writing the manuscript.

## Conflict of Interest

The authors declare that the research was conducted in the absence of any commercial or financial relationships that could be construed as a potential conflict of interest.

## Footnotes

^1^http://dx.doi.org/10.18150/repod.0107441

^2^Notice that the connectivity measures are not *symmetric* and hence GPDC_*ij*_(*f*)≠GPDC_*ji*_(*f*).

^3^This is simply an independent draw from a distribution, which is here provided by the training dataset.

^4^Notice that, since we have 14 healthy subjects and 14 patients, this scheme yields perfectly balanced (same number of cases and controls) training and test sets.

^5^There is now a trade-off between specificity and sensitivity, but it is still possible to get a useful classifier.
